# Differences in Weekly Training Load, Well-Being, and Hormonal Responses between European- and National-Level Professional Male Basketball Players during the Pre-Season Phase

**DOI:** 10.3390/ijerph192215310

**Published:** 2022-11-19

**Authors:** Daniele Conte, Paulius Kamarauskas

**Affiliations:** Institute of Sport Science and Innovations, Lithuanian Sports University, Sporto St. 6, LT-44221 Kaunas, Lithuania

**Keywords:** salivary testosterone, salivary cortisol, RPE, preparation period

## Abstract

This study aimed to compare the weekly fluctuation in training load (sRPE-load), well-being (perceived fatigue, stress, mood, sleep, and muscle soreness), and hormonal responses [testosterone (T) and cortisol (C)] during the pre-season phase in European- and national-level professional male basketball players. Twenty-one professional male basketball players belonging to European-level (n = 11, age: 25.5 ± 3.6 yr; stature: 199.2 ± 7.1 cm; body mass: 94.1 ± 8.5 kg) and national-level (n = 10, age: 23.5 ± 4.7 yr; stature: 198.0 ± 5.6 cm; body mass: 94.0 ± 8.8 kg) teams were monitored during a 5-week pre-season phase. The European-level team showed higher sRPE-load in week 5 compared to week 3 (within-team difference, *p* = 0.049; ES = −1.44 [−2.38, −0.5], large) and week 1 (*p* = 0.018; ES = 1.62 [0.64, 2.61], large), week 4 (*p* = 0.005; ES = 1.79 [0.78, 2.81], large) and week 5 (*p* = 0.001; ES = 1.96 [0.92, 3.01], large) of the national-level team. The national-level team documented the lowest sRPE-load in week 5, which was statistically different compared to week 2 (*p* = 0.022; ES = 1.59 [0.61, 2.58], large) and week 4 (*p* = 0.001; ES = 1.94 [0.9, 2.98], large) of the European-level team. Moreover, higher stress (*p* < 0.001; ES = 1.94 [0.93, 2.95], large) and better mood (*p* = 0.003; ES = 1.79 [0.8, 2.78], large) were found in week 1 compared to week 5 within the European-level team. Additionally, higher values of salivary C were found in week 1 for the European-level team compared to week 2 (*p* = 0.020; ES = 1.6 [0.61, 2.58], large) and week 4 (*p* = 0.018; ES = 1.66 [0.64, 2.67], large) of the national-level team. Our results can provide reference values for basketball practitioners regarding the fluctuations of weekly load, well-being, and hormones across the pre-season period in professional male teams competing at European and national levels.

## 1. Introduction

The pre-season phase is a fundamental part of the basketball season aiming at increasing players’ physiological and physical characteristics for the upcoming in-season phase. In this part of the season, designing appropriate training sessions with sound periodization strategies is essential to achieve optimal performance outcomes and reduce the possibility of non-functional overreaching, overtraining, and injuries [1]. Therefore, monitoring the training load experienced by basketball players during this period is important to assess whether the prescribed load was adequate to obtain an enhancement of players’ physiological and physical performances.

Previously, it has been indicated that the pre-season phase is characterized by a higher training volume compared to the in-season phase due to the higher number of training sessions and the absence of official matches [2]. Moreover, the workload experienced by basketball players is based on their competitive levels. Indeed, it has been shown that professional basketball players accumulate approximately double the training load assessed via the weekly session rating of perceived exertion (sRPE-load) compared to their semi-professional counterparts (5241 ± 1787 vs. 2408 ± 487 AU [3] and 5058 ± 1849 vs. 2373 ± 488 AU [4]. It should be noted that this training load difference has been assessed only between teams competing in different competition levels across higher or lower division leagues (1st and 2nd division Italian league vs. 3rd division league) [3,4], while a difference might also exist between teams involved in the same professional league. In fact, European professional basketball leagues are composed of European-level teams, which are usually competing in both national and European (e.g., Euroleague, Eurocup) championships, and national-level teams, which compete in national leagues only. Therefore, these teams are characterized by a different match schedule, with European-level teams being involved in a higher number of official matches compared to national-level teams across the season. While the congested match schedule has been shown to determine a different workload across the in-season phase in collegiate [5] and semi-professional [6,7] male basketball players, no information is available on the pre-season phase. Indeed, the adaptation needed by athletes competing in a less or more congested match schedule might vary following different periodization strategies. Therefore, monitoring the pre-season training sessions completed by professional teams competing at the European- and national-level is warranted. 

Monitoring the workload experienced during the pre-season phase provides only partial information about the adaptation of basketball players. Differently combining workload with other monitoring tools, such as well-being measured with self-reported scales and hormonal responses such as salivary cortisol (C) and testosterone (T), could provide a clearer picture of players’ adaptations to the imposed load [6]. Overall, monitoring these parameters can provide a better understanding of players’ training and recovery process [8]. Despite the importance of monitoring these measures during the pre-season phase, only a few studies previously provided indications about the well-being and C and T levels encountered by professional male basketball players during the pre-season phase [9,10]. Although these studies provide important information regarding the fluctuation of these parameters across the pre-season period, no information is available on the changes in these measures in professional male basketball teams competing at European- or national-level. This information might be relevant for basketball practitioners to have a better understanding of the training and recovery process experienced by professional male players, possibly across different periodization strategies, competing in the same league but possessing different upcoming in-season schedules. Therefore, the aim of this study was to assess and compare the weekly fluctuation in training load, well-being, and hormonal responses during the pre-season phase in European- and national-level professional male basketball players.

## 2. Materials and Methods

### 2.1. Participants

Twenty-six male basketball players from two professional teams competing at European- (FIBA EuroCup and first-tier Lithuanian basketball league [Lietuvos krepšinio lyga, LKL]), and national-level team (only LKL) participated in this study. Five players (European-level team, n = 2; national-level team, n = 3) with less than 75% attendance were excluded from the analysis. Therefore, the final study consisted of 21 professional male basketball players (Table 1). An a priori analysis indicated that the present study was sufficiently powered (α = 0.05; β = 0.80; effect size = 5.56) (G*Power, version 3.1; University of Dusseldorf, Dusseldorf, Germany) based on a previous investigation assessing the difference in sRPE-load between professional and semi-professional basketball players [3]. Prior to the monitoring period, all participants were instructed and familiarized with the purpose, procedures, requirements, risks, and benefits of the investigation and provided written consent of participation. Ethical approval was obtained from the Kaunas Regional Research Ethical Committee review board (No. BE-2-97).

### 2.2. Design

The data for this observational study was collected during the 5-week of the 2020/21 pre-season phase (European-level team: 10 August–13 September 2020; national-level team: 11 August–15 September 2020). This study investigates a new research question, but it should be noted that part of this dataset (i.e., data of European-level team) was previously examined in another investigation with different research questions [9]. During the monitoring period, players’ training load was collected across 37 training sessions and 5 pre-season matches (3 wins by 35.3 ± 18.2 points; 2 losses by 13.0 ± 15.6 points) for the European-level team and across 42 training sessions and 5 pre-season matches (3 wins by 22.7 ± 19.1 points, 2 losses by 23.0 ± 7.1 points) for the national-level team (Table 2). Overall, the final sample consisted of 429 and 444 training and friendly match samples for the European- and national-level teams, respectively. Well-being measures were collected daily, while saliva samples were collected weekly from the end of the first week of the pre-season phase for both teams.

### 2.3. Procedures

Training load was measured using sRPE-load, which has been extensively adopted in basketball [5,11,12,13,14]. About 30 min following each training session and friendly match, players were required individually by the research team to provide their scores using the modified Borg 10-point sRPE scale (CR-10) [14]. sRPE data were then multiplied by the session duration in minutes (including warm-up, breaks, and stoppage in play during friendly matches) to obtain the sRPE-load value. Weekly sRPE-load was calculated as the sum of weekly values. Data were collected and stored on cloud-based software (Google Docs, Microsoft, CA, USA). The compliance of sRPE was 91% and 97% for the European- and national-level teams, respectively. 

Players’ well-being was measured using a questionnaire previously used in basketball literature [5,6,12,13]. Every morning before 10:00 a.m., the research team administered the well-being questionnaire for each player to evaluate his perceived level of fatigue, sleep quality, general muscle soreness, stress, and mood on a five-point Likert scale (scores from 1–5) [5,13]. Data were collected and stored in cloud-based software (Google Docs, Microsoft, CA, USA). The total well-being score was calculated by summing the scores across each item assessed [5,13]. For the final analysis, weekly values were averaged for each item and for total well-being. The compliance of well-being was 99% and 95% for the European- and national-level teams, respectively.

Salivary testosterone T, C, and their ratio (T:C) were used to monitor players’ hormonal responses. A member of the research team collected saliva samples weekly across the pre-season phase (i.e., 5 sample collections), prior to the first training session of the week, at the same time of the day (17:00), to avoid variation due to circadian rhythm [15]. Players were instructed not to eat, brush their teeth, and drink only still water in the 90min prior to saliva collection [6,9]. Successively, players were required to mouth rinsing with distilled water and remove all saliva from the mouth. Then, they were asked to remove all saliva again after 30 s, to wait in a seated position for 10 min, and to provide the saliva specimen into 15-mL SaliCap tubes through a polypropylene straw (IBL International, Germany [6,9]. The collected samples were stored in a laboratory at −20 °C for subsequent analysis. T and C were determined in duplicate using an enzyme-linked immunoassay (IBL International, Germany) following manufacturer instructions (REF for T: RE52631; REF for C: RE52611). The intra-assay coefficient of variation for T and C analyses were 2.51% and 3.22% for the European-level team and 2.19% and 3.09% for the national-level team, respectively.

### 2.4. Statistical Analysis

Mean and SD were calculated as descriptive statistics. Linear mixed models (LMMs) were used to assess the differences between weeks, teams, and their interaction for all dependent variables (i.e., sRPE-load, fatigue, sleep, soreness, stress, mood, well-being, T, C and T:C), using week and team as a fixed effect, and players as a random effect. All random effects were considered with random intercept and fixed slope. The assumption of normality for residual values was checked using the Kolmogorov–Smirnov test. Post-hoc analysis with Bonferroni correction was used in case of significant differences. All LMMs were analyzed using Jamovi software (the Jamovi project, version 2.3.3, 2022, Sydney, Australia, which was previously used in basketball research [9,16]. Available online: https://www.jamovi.org). An alpha level of *p* < 0.05 was set at a priori for statistical significance. Cohen’s d effect sizes were also calculated for statistically significant post-hoc analyses using the t-value of each comparison using the “compute.es” package on the RStudio software (Version 1.0.153 for Windows) and interpreted as follows: trivial ≤ 0.20; small = 0.20–0.59; moderate = 0.60–1.19; large = 1.20–1.99; and very large ≥ 2.0 [17].

## 3. Results

**Figure 1:**
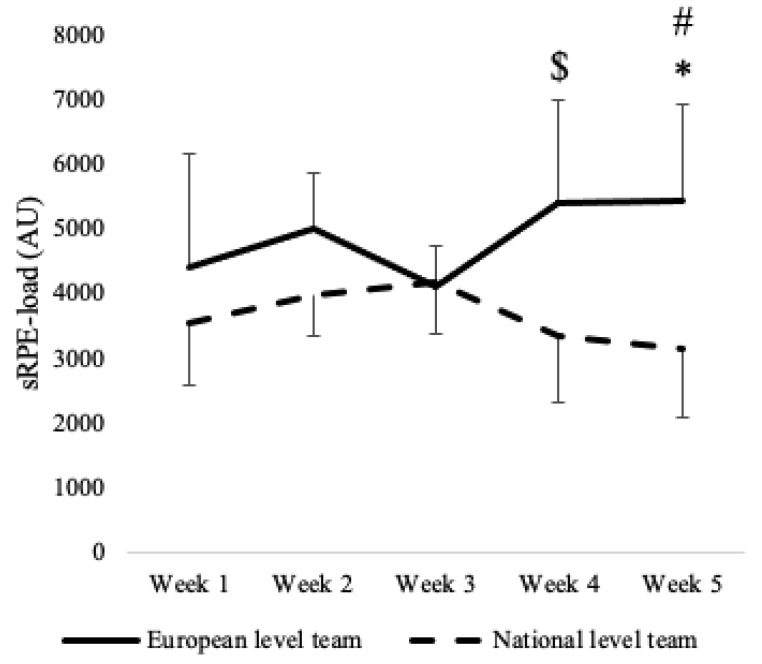
Weekly values (mean ± SD) and post-hoc analysis in sRPE-load. Note. ^$^ significant difference compared to week 1 (*p* = 0.022), week 4 (*p* = 0.006), and week 5 (*p* = 0.001) of national-level team; ^#^ significant difference compared to week 3 (*p* = 0.049) of European-level team, and week 1 (p = 0.018), week 4 (*p* = 0.005), and week 5 (*p* =0.001) of national-level team; * significant difference compared to week 2 (*p* = 0.022) of European-level team.

A team effect was found for sRPE-load with higher values registered for the European-level team compared to the national-level team (*p* = 0.002), while no effect of week was found (*p* = 0.376) (Figure 1). Furthermore, a significant interaction between team and week was found (*p* < 0.001). Post-hoc analyses showed the highest sRPE-load value for the European-level team in week 5, with within-team differences revealed in week 3 (*p* = 0.049; ES = −1.44 [−2.38, −0.5], large). Moreover, the European-level team in week 5 showed higher sRPE-load compared to week 1 (*p* = 0.018; ES = 1.62 [0.64, 2.61], large), week 4 (*p* = 0.005; ES = 1.79 [0.78, 2.81], large) and week 5 (*p* = 0.001; ES = 1.96 [0.92, 3.01], large) of the national-level team. Additionally, the national-level team documented the lowest sRPE-load in week 5, which was statistically different compared to week 2 (*p* = 0.022; ES = 1.59 [0.61, 2.58], large) and week 4 (*p* = 0.001; ES = 1.94 [0.9, 2.98], large) in the European-level team. Finally, the European-level team showed a higher sRPE-load in week 4 compared to the national-level team in week 1 (*p* = 0.022; ES = 1.6 [0.61, 2.58], large) and week 4 (*p* = 0.006; ES = 1.76 [0.76, 2.77], large). 

**Figure 2:**
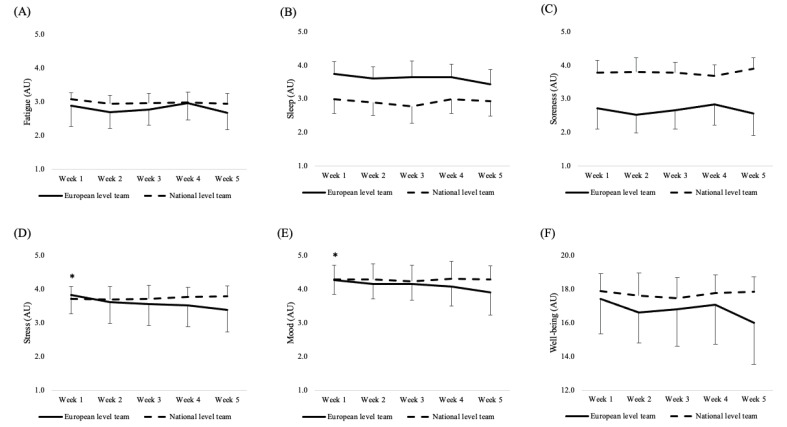
Weekly values (mean ± SD) and post-hoc analysis in (**A**) fatigue; (**B**) sleep; (**C**) soreness; (**D**) stress; (**E**) mood; (**F**) well-being. Note. * significant difference compared to week 5 (stress, *p* < 0.001; mood, *p* = 0.003) within the European-level team.

In considering total well-being, results revealed no effect of team (*p* = 0.170) week (*p* = 0.053) and their interaction (*p* = 0.067), while some statistical differences were found when considering well-being items singularly (Figure 2). Specifically, a team effect was found for sleep (*p* < 0.001) and general muscle soreness (*p* < 0.001). Moreover, while no effect of team and week was found, an interaction was evident for stress (*p* = 0.005) and mood (*p* = 0.029). Post-hoc analyses showed higher stress (*p* < 0.001; ES = 1.94 [0.93, 2.95], large) and a better mood (*p* =0.003; ES = 1.79 [0.8, 2.78], large) in week 1 compared to week 5 within the European-level team (Figure 2). 

**Figure 3:**
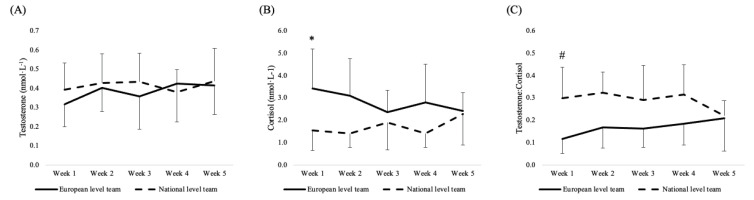
Weekly values (mean ± SD) and post-hoc analysis in (**A**) testosterone; (**B**) cortisol; (**C**) testosterone: cortisol ratio. Note. * significant difference compared to week 2 (*p* = 0.02) and week 4 (*p* = 0.018) of national-level team; ^#^ significant difference compared to week 1 (*p* = 0.011), week 2 (*p* = 0.002), week 3 (*p* = 0.018) and week 4 (*p* = 0.003) of national-level team.

The analysis of hormonal responses revealed no effect of team and week on salivary T (*p* > 0.05), while a team effect was found for C (*p* = 0.004) and T:C (*p* < 0.001), respectively (Figure 3). C and T:C also showed an interaction effect (*p* = 0.026 and *p* = 0.013, respectively). Post-hoc analyses revealed higher values in C level in week 1 for the European-level team compared to the values registered in week 2 (*p* = 0.020; ES = 1.6 [0.61, 2.58], large) and week 4 (*p* = 0.018; ES = 1.66 [0.64, 2.67], large) for the national-level team. Moreover, the analysis of T:C indicates a higher value for the national-level team in week 1 compared to the values in week 1 (*p* = 0.011; ES = −1.73 [−2.76, −0.7], large), week 2 (*p* = 0.002; ES = −1.89 [−2.92, −0.86], large); week 3 (*p* = 0.018; ES = −1.62 [−2.6, −0.63], large) and week 4 (*p* = 0.003; ES = −1.89 [−2.95, −0.83], large) of the European-level team (Figure 3). 

## 4. Discussion

This study aimed to assess and compare the weekly training load, well-being, and hormonal responses during a 5-week pre-season phase in European- and national-level professional male basketball teams. The main findings revealed a different periodization strategy adopted by the two teams, with the European-level team reporting an increasing training load toward the end of the pre-season, while the national-level team showed a decreasing load. This outcome did not induce differences over time for both teams in total well-being and T levels, while the European-level team showed overall higher C and lower T:C levels across the pre-season period.

Previous studies comparing the training load experienced during the pre-season phase by teams with different competitive levels (professional vs. semi-professional) showed higher weekly sRPE-load for the higher-level team (~5000 AU) compared to lower-level teams (~2400 AU) due to the higher amount of training sessions performed by the professional team [3,4]. Our results are in line with these previous investigations [3,4] since the European-level team show significantly higher weekly sRPE-load (4877 ± 1390 AU) compared to the national-level team (3645 ± 950 AU). These results were surprising since the European-level team was involved in less training and fewer friendly match sessions (37 training and 5 friendly matches) compared to the national-level team (42 training and 5 friendly matches). This difference could be explained by the discrepancy in session durations for the two investigated teams. Indeed, the European-level team had training sessions with longer duration, averaging 115 min per session per week, compared to the national-level team (88 min per session per week). Since the sRPE-load is an internal load measure directly depending on training and match durations, the longer sessions of the European-level team might be the reason for this result.

Another interesting consideration regards the different periodization strategies adopted by the two investigated teams. Indeed, the national-level team showed a training schedule encompassing a decrease in the number of training sessions from the first to the last pre-season week (week 1: 12 sessions; week 2: 10 sessions; week 3: 10 sessions; week 4: 8 sessions; week 5: 7 sessions), which led to a reduction of sRPE-load toward the end of the pre-season phase. In contrast, the European-level team showed a more undulating training schedule with the last week, including the lowest number of training sessions (week 1: 9 sessions; week 2: 10 sessions; week 3: 7 sessions; week 4: 9 sessions; week 5: 7 sessions). Interestingly, although players had a similar number of training sessions in the last two weeks, the European-level team showed the highest sRPE-load values, while the national-level team showed the lowest sRPE-load values. A possible reason for this result could be that in the last weeks of the pre-season period, the European-level team coaching staff included friendly matches played with short in-between recovery time (i.e., two matches played on consecutive days in week 5), trying to provide a scenario similar to the upcoming in-season phase characterized by a congested match schedule. In comparison, the national-level team scheduled friendly matches at least three days apart, which is also a typical scenario for teams involved only in domestic leagues. Moreover, the higher sRPE-load experienced by the European-level team at the end of the pre-season phase compared to the national-level team is also due to the longer duration of friendly matches (including warm-up and stoppage times) compared to training sessions and friendly matches of the national-level team. Previous literature showed an alteration of the internal and external load due to a different match schedule in collegiate [5] and semi-professional [6,7] male basketball players. To the best of our knowledge, this is the first investigation assessing the load experienced by male professional basketball players in preparation for an in-season phase with a different number of weekly matches, which indicates the novelty of this research. Nevertheless, it should be noted that we collected training load only via sRPE-load, which was a subjective measure of internal load, while no information was available about the external load accumulated during the monitored period, making it hard to have full consideration of the prescribed load and therefore the periodization strategy adopted by both teams.

Different from load data, players’ perceived well-being was similar within- and between teams. However, a *p*-value close to significance (*p* = 0.053) was found for the week, highlighting a tendency in difference across weeks for both teams. Graphically, it is possible to notice a decrease in week 5 in total well-being compared to previous weeks in the European-level team (Figure 2). A possible reason for this decrease, although not statistically significant, might be due to the sRPE-load values recorded in week 5, which indicated the highest value across the monitored period for the European-level team. Overall, this outcome might indicate a likely negative correspondence between the sRPE-load and well-being values. However, previous literature documented a negative, small (rho = −0.150) relationship between sRPE-load calculated during matches performed across a European championship in youth female basketball players, and the well-being measured in the following morning [18]. Overall, considering our outcomes and those reported in previous literature [18], it seems important for basketball practitioners to monitor both load and well-being across various parts of the season due to their small relationship. It also seems important to investigate further the relationships between load and well-being across different times of the seasons and different basketball populations, which might provide useful indications for practitioners.

The importance of monitoring players’ well-being in combination with training load during the pre-season phase in professional male basketball players is also substantiated by the results reported when considering well-being items singularly. Indeed, European-level teams showed higher muscle soreness across the considered pre-season phase compared to the national-level team. Furthermore, better stress and mood levels were found in week 1 compared to week 5 for the European-level team, while they were constant for the national-level team. Although these psychological measures have multifactorial components, one of the possible reasons for these results might be the load perceived by players and the possible periodization strategy adopted by the coaching staff. Indeed, the higher muscle soreness reported by European-level players might be due to the higher load perceived across the pre-season phase compared to national-level teams. Similarly, the increased perceived load found in the European-level team toward the end of the pre-season can also be a potential reason for the decrease in mood and increase in stress documented across the same timeframe. Our results are similar to those reported in the literature. Indeed, Nunes et al. [15] showed higher stress levels in response to an increased sRPE-load during a 12-week periodized training program in elite female basketball players. Additionally, Miloski et al. [19] showed a decreased mood state in response to the 5-week overloading phase in young male basketball players. Overall, our results suggest a possible sensitivity of these psychological measures assessed individually in relation to sRPE-load across the pre-season phase in professional male basketball players.

The analysis of hormonal responses showed no effect of team and week in T levels, while overall higher levels of C were found in the European-level team compared to the national-level team across the investigated period. The post-hoc analyses revealed higher C levels for European-level players at the end of the first week compared to week 2 and week 4 registered by national-level players. Additionally, when considering T:C, lower values in European-level players were found in week 1 compared to week 1, week 2, week 3, and week 4 in national-level players. These outcomes suggest the dominance of the catabolic processes and a deteriorated recovery [20,21], or potentially, the difference in individual training status with lower player readiness (i.e., low fitness and high fatigue) [22] for the European-level team compared to the national-level team at the beginning of the monitored period. This difference might also be due to the different training typologies adopted by the two investigated teams. In fact, while the national-level team used only strength and conditioning sessions, the European-level coaching staff prescribed a combination of strength and conditioning and technical and tactical sessions characterized by a longer duration. Potentially, the combination of long court-based basketball sessions and strength and conditioning sessions might have been felt as a potential stressor and increased the catabolic processes in European-level players. However, it has hard to have a full understanding of the mechanisms behind the C changes across the pre-season phase. In fact, it should be noted that no data on the beginning of the pre-season were available in the current study, and the period preceding the pre-season period (i.e., off-season) was not monitored, making hard to have a full understanding of players’ baseline levels from a hormonal standpoint. Moreover, the hormonal responses cannot be associated or anticipated by the workload (either internal or external), as previously shown in professional male basketball players during the pre-season phase [9]. Indeed, not only physical and physiological but also psychological factors can play an important role in players’ C and T responses during the pre-season [9]. Therefore, future investigations are necessary to fully understand the mechanisms related to changes in hormonal responses during the pre-season phases in basketball players. Overall, it seems important to monitor C and T, which can still provide important information for basketball coaches regardless of the physical or physiological stress imposed.

This investigation provides some novel findings about the differences between professional male basketball players preparing to compete in the different levels of championships, but there are some limitations that should be considered. Firstly, only the measures of subjective internal load, well-being, and hormonal responses were assessed during the investigating period, while the inclusion of objective internal and external load measures could provide a more accurate snapshot of the load differences in male professional basketball teams at the national and European levels. Therefore, future studies should include objective external and internal load measures to provide more detailed results.

## 5. Practical Applications

Our results provide an indication for basketball practitioners and coaches about the possible periodization strategies adopted by the coaching staff of male professional basketball teams competing at the national and European levels. Moreover, we provide an indication of the well-being and hormonal responses encountered during the pre-season phase in male professional basketball, which are similar in most of the investigated measures while differing across time in stress, mood, and between teams in C and T:C levels. Furthermore, it suggests monitoring load, well-being, and hormonal responses separately since each measure specifically fluctuates across the investigated period in the two teams. Finally, these results can provide reference values for basketball coaches and practitioners monitoring subjective internal load, well-being, and hormonal responses in male professional basketball players during the pre-season phase. 

## 6. Conclusions

In conclusion, our investigation revealed a different perceived load across the pre-season period in male European- and national-level teams, possibly due to different periodization strategies adopted in preparation for the upcoming season, with an increased load for the European-level team and a decreased load for the national-level team at the end of the pre-season period. These differences elicited increased perceived stress and lower mood at the end of the pre-season period compared to the first week in the European-level team, while no within-team changes were found for the national-level team. However, no between- and within-team differences were found in total well-being. Moreover, the different periodization strategies and exercise typologies likely produced different hormonal responses, with the European-level team revealing higher C and lower T:C values in the first training week compared to the following weeks of the national-level team. Overall, our results can provide reference values in terms of load, well-being, and hormonal responses in professional male basketball players during the pre-season phase, and considering the specific fluctuations of each load, well-being measure, and hormonal response suggested the monitoring of all these measures in combination. 

## Figures and Tables

**Table 1:** Characteristics of participants.

	European-Level	National-Level
Sample (n)	11	10
Age (yr)	25.5 ± 3.6	23.5 ± 4.7
Stature (cm)	199.2 ± 7.1	198.0 ± 5.6
Body mass (kg)	94.1 ± 8.5	94.0 ± 8.8

Note: Data presented as mean ± standard deviation.

**Table 2:** Pre-season phase schedule of European- and National-level teams.

			European-Level Team								National-Level Team						
			Day1	Day2	Day3	Day4	Day5	Day6	Day7		Day1	Day2	Day3	Day4	Day5	Day6	Day7
Week 1	Morning	Typology	-	PT	-	SC, SH	SC	SC, FB	-		PT	SC	SC	SC	-	SC	SC
		Duration (min)		112		127	104	81			45	65	71	90		80	70
	Afternoon	Typology	SC	SC	SC, BB	BB	BB	-	-		SC	SC	SC	SC	-	SC	SC
		Duration (min)	78	109	109	118	104				76	82	65	45		82	70
Week 2			Day1	Day2	Day3	Day4	Day5	Day6	Day7		Day1	Day2	Day3	Day4	Day5	Day6	Day7
	Morning	Typology	SC, SH	SC	SC, BB	SC, SH	SC, SH	SC, FB	-		SC	SC	-	SC, SH	SC, SH	-	SC
		Duration (min)	90	97	130	101	120	83			65	45		115	76		60
	Afternoon	Typology	BB	BB	-	BB	BB	-	-		SC	-	BB	SC	BB	-	BB
		Duration (min)	130	103		112	120				85		100	110	135		135
Week 3			Day1	Day2	Day3	Day4	Day5	Day6	Day7		Day1	Day2	Day3	Day4	Day5	Day6	Day7
	Morning	Typology	SC, SH	-	-	SC, SH	-	SC, BB	-		SC, SH	SH	SC, SH	SC, SH	-	-	SC
		Duration (min)	118			102		109			135	69	60	90			60
	Afternoon	Typology	BB	BB	-	BB	FM	-	-			FM	BB	BB	FM	-	BB
		Duration (min)	130	106		116	178					158	90	88	143		90
Week 4			Day1	Day2	Day3	Day4	Day5	Day6	Day7		Day1	Day2	Day3	Day4	Day5	Day6	Day7
	Morning	Typology	SC, SH	BB	SC, BB	-	BB	-	SC, BB		SC	SC, BB	SC, BB	-	-	-	SC
		Duration (min)	113	55	110		50		130		60	80	60				60
	Afternoon	Typology	BB	FM	-	BB	FM	-	-		BB	-		FM	-	BB	BB
		Duration (min)	106	187		95	194				90			132		125	90
Week 5			Day1	Day2	Day3	Day4	Day5	Day6	Day7		Day1	Day2	Day3	Day4	Day5	Day6	Day7
	Morning	Typology	-	-	-	PT	SC, BB	-	-		BB		-	SC	BB	-	PT
		Duration (min)				118	110				90			60	90		60
	Afternoon	Typology	FM	FM	-	-	BB	BB	BB		-	FM	-	BB	-	FM	-
		Duration (min)	189	211			120	95	90			115		105		128	

Note. SC—strength and conditioning session; PT—performance testing session; BB—basketball training sessions (i.e., technical, tactical); FM—pre-season friendly match; FB—football (soccer) session; SH—shooting session.

## Data Availability

Not applicable.
